# Gossypiboma in the oral region: Case report and literature review

**DOI:** 10.4317/jced.51553

**Published:** 2014-10-01

**Authors:** Camila N. Alves-de-Oliveira, Tania-Mara Pimenta-do-Amaral, Giovanna Ribeiro-Souto, Ricardo Alves-Mesquita

**Affiliations:** 1 Department of Oral Pathology and Surgery. School of Dentistry. Universidade Federal de Minas Gerais. Faculdade de Odontologia. Brasil

## Abstract

Gossypiboma is an inflammatory reaction to a foreign body, specifically composed of a cotton matrix left behind after surgery. The present study aims to present a case report of gossypiboma 23 years after a dental surgery and to make a literature review of the English language cases published on the issue. A 42-year-old woman was attended to evaluate complaints of pain on the right side of the maxilla. The patient reported that dental extractions in this region had been performed over a period of nearly 23 years. The panoramic radiograph demonstrated an opacity in the right maxillary sinus, which presented a spongiform aspect, irregularly shaped radiopaque image, as well as a rupture of the maxillary sinus’s lower cortical layer. A surgical excision was performed, and the histopathological diagnosis was of gossypiboma. Six similar cases have also reported of gossypiboma in the oral region; however, calcification was only identified in the present case report. The patient is currently undergoing follow-up and has presented no complaints of pain or recurrence.

** Key words:**Gossypiboma, textiloma, muslinoma, gauzoma, oral region.

## Introduction

Gossypiboma is derived from the Latin “gossypium”, the genus of cotton plant types and from boma, a Swahili word meaning “place of concealment.” This term is applied to the inflammatory response caused by the introduction of a foreign body (1). Gossypiboma has also been labeled in prior literature as textiloma, cottonoid, gauzoma, or muslinoma, which are rare conditions caused solely by iatrogenic factors. However, a gauze left behind after surgery is a serious medical/legal issue and is thus often under-reported in the literature (2,3).

Physical surgeries with large openings, including abdominal, otorhinolaryngology, plastic, orthopedic, and cardiothoracic surgeries, have all reported cases of gossypibomas (4,5). Prior literature has reported a 76% incidence of a false count of surgical sponges after having completed the surgical procedures, while an incidence of gossypiboma varies between 1/1000 and 1/10000 (2). Most commonly found in the literature are cases of gossypiboma associated with neurosurgical procedures in the head and neck region, with only a few reported cases associated with oral surgery. A search in the annals of PUBMED for gossypiboma, textiloma, cottonoid, gauzoma, or muslinoma in oral region, as well as in the maxillary sinus, revealed six similar cases (4-7). Fever, swelling, tenderness, and purulent discharge from an oral wound after surgery may indicate the presence of gossypiboma (6). Some patients are either asymptomatic or take a very long time to present any symptoms of inflammation or fistula formation. Diagnosis is determined both by the association of clinical and imaging exams as well as by surgical removal, in which the gauze left behind can be identified. Radiographic or computed tomography [CT] features of oral gossypiboma may vary among radiolucent, isodense, low-density, or heterogeneous masses, depending on the type of reaction produced by the body due to the gauze (2,5). Ultrasound analysis shows a hypoechogenic mass associated with the hyperechogenic region (6).

The present study aims to report on a case of gossypiboma in the maxillary sinus after a 23-year period of tooth treatment and extraction, as well as presents a literature review of case reports in the English-language concerning gossypiboma in the oral region.

## Case Report

**Figure 1:**
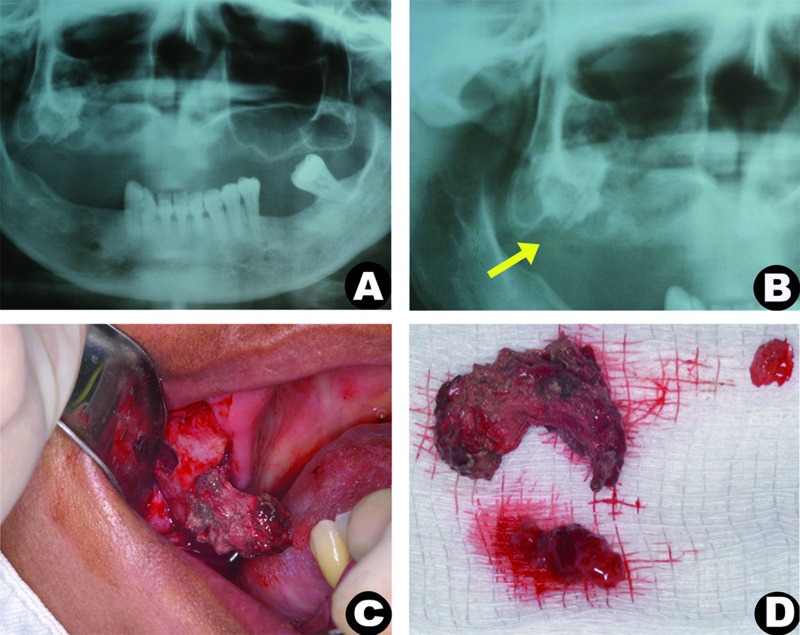
(a) Panoramic radiograph: showing radiopaque image in left maxillary sinus; (b) The lesion and the rupture of the lower cortical layer (yellow arrow) can be seen in greater detail; (c) intraoperative procedure demonstrates a removal of a well-defined lesion; (d) and the major specimens presented an oval shape.

A 42-year-old woman was attended to evaluate complaints of pain on the right side of the maxilla. The patient’s medical history included hypertension, gastric ulcers, dizziness, and fainting, all controlled with medication. Extraoral examination revealed no abnormalities. The patient reported pain in the maxilla, which had begun two years ago and which had become more intense in the last two months. This pain was accompanied by a pus secretion from the lesion, which often presented a foul smelling secretion within the nasal discharge. Nearly 23 years before, the patient had undergone dental extractions in this same dental region, producing a complication within the maxillary sinus that had not been previously addressed. Intraoral evaluation revealed an edentulous maxilla with a fistula in the right molar region, which presented a purulent, foul-smelling drainage. The patient does not use removable upper dentures, but pain was reported during palpation. The panoramic radiograph presented an opacity on the right maxillary sinus and a homogeneous, slightly circular with ill-defined borders, radiopaque image, measuring approximately 2.0 x 2.5 cm. In addition, a rupture of the lower cortical layer could also be observed (Fig. 1).

The differential clinical-radiographic diagnosis included either inflammatory or infectious processes, such as chronic sinusitis caused by oroantral perforation, the formation of fungus balls [mycetoma or aspergillosis], the presence of a foreign body, or large antroliths. Cementoblastoma, complex odontoma, calcifying epithelial odontogenic tumors, fibrous dysplasia, calcifying cystic odontogenic tumors, and osteosarcoma were found to be less possible options.

**Figure 2:**
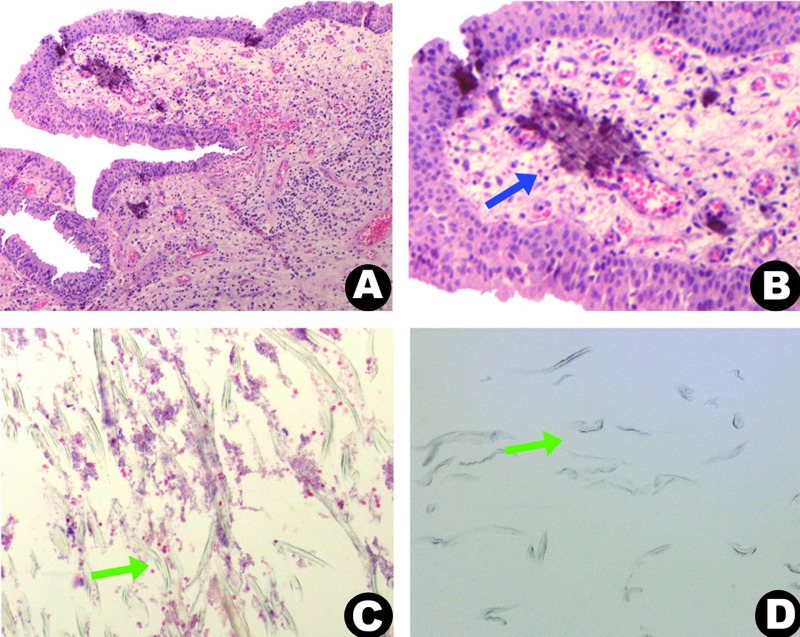
(a) Microscopic features demonstrate respiratory epithelium with inflammatory infiltrate; (b, blue arrow) and calcification; (c, green arrow) Figure display linear, angular, and sometimes birefringent structures; (d, green arrow) that can be observed in the gossypiboma; note the similar structures in the microscopic view of the lesion and in the inclusion of gauze.

Surgical exploration for the biopsy was performed. However, because of the intraoperative condition, in which the lesion was well-defined and could be easily excised, the lesion was surgically removed (Fig. 1). A fibrin sponge was placed inside the maxillary sinus to avoid bleeding and to facilitate the closure of the incision. The surgical specimens (Fig. 1) were sent for histopathology analysis. The largest fragment was hard and had undergone decalcification. Microscopic analysis demonstrated an epithelial lining of the respiratory tract, loose connective tissue with chronic inflammatory cells, and dystrophic calcification (Fig. 2). The decalcified fragment appeared as numerous, eosinophilic, linear structures, which were at times angular and birefringent (Fig. 2). Staining for PAS and Gomori-Grocott proved to be negative. Considering the possibility that these structures represent an exogenous material made of gauze, a sample of gauze was processed and included in laboratory. The image of the included gauze proved to be identical to the previously described material (Fig. 2).

In follow-up, the patient was again questioned about her prior surgeries. Was reported that they had been quite traumatic and that had to return to the dentist repeatedly to stop the bleeding and put this “thing” [as the patient called it] in place of the extracted teeth. This information made it clear that the proper diagnosis was of gossypiboma.

**Figure 3:**
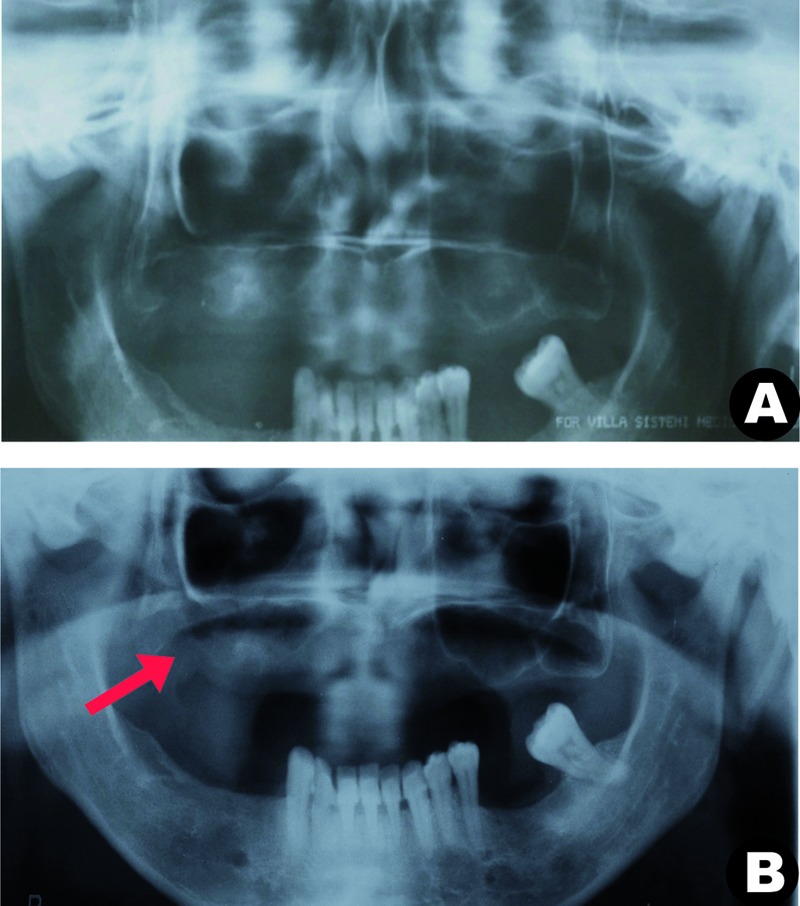
(a) Panoramic radiograph 15 days after surgery; (b, red arrow) showed a partial opacity of the maxillary sinus, compatible with inflammation in the region in remission, and follow-up after 4 months, where a thin radiopaque line of the lower cortical layer of the maxillary sinus can be observed.

A panoramic radiograph was taken 15 days after this final surgical procedure. No remnant of the gauze could be observed. What could be observed was a partial opacity of the maxillary sinus, which was compatible with the inflammation in the region (Fig. 3). The patient subsequently underwent the surgical correction of oroantral communication, and in follow-up, the patient reported no complaints of pain or recurrence (Fig. 3).

## Discussion

A review of the English language literature in the PUBMED database was carried out on case reports of gossypiboma in the oral region and the maxillary sinus, using the following keywords: gossypiboma, textiloma, muslinoma, and gauzoma. Six cases were found, as shown in  Table 1.

**Table 1:**
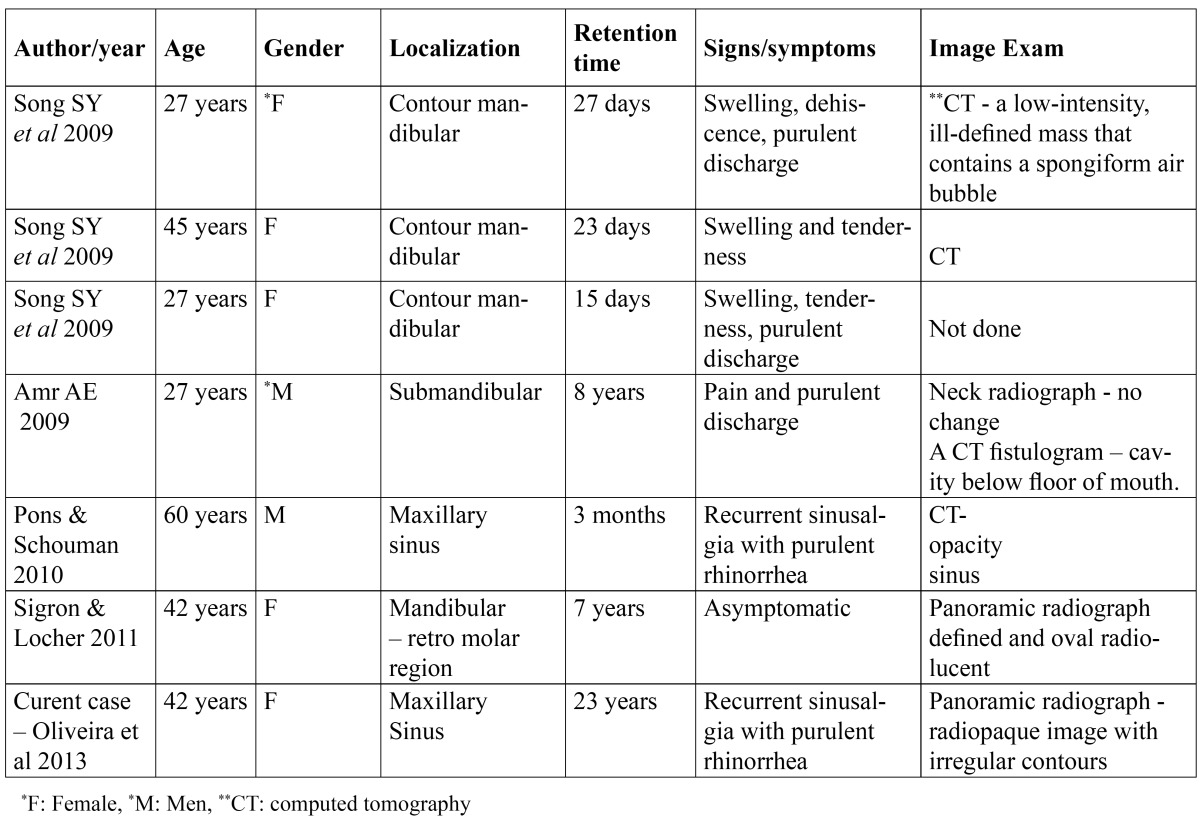
Summary of gossypiboma in oral region and maxillary sinus reported in English-language literature.

The patients ranged between 27 and 60 years of age, and were predominantly female. Three cases of gossypiboma were reported after having undergone plastic surgery of the mandibular contour (6), one case in the submandibular region after the right submandibular gland had been removed (7), another after extraction of the third molar from the retromolar region (5), and yet another in the maxillary sinus (4). The maxillary sinus case developed after having removed a cyst form the maxillary sinus using the Caldwell-Luc approach. In the current case report, the gossybipoma proved to be related to tooth extraction.

The formation of gossypiboma is directly linked to iatrogenesis in surgical procedures, in such a way that the chances of identifying a gossypiboma after major surgery are higher, since the gauze used are larger in both size and number. Swelling and improper closing of the surgical wound, with drainage of a purulent discharge, are indicative of inflammation or infection, which may not be related to the presence of a foreign body. Song *et al*. [2009] (6) cited 2 types of reactions. One is an aseptic fibrinous response that results in adhesion or encapsulation, in turn leading to the formation of granulomas. The other is an exudative-type formulation leading to an abscess, with or without bacterial superinfection. In the current case, the presence of chronic inflammation with purulent drainage could be identified, thus indicating the exudative-type formulation.

Considering all the cases, the longest time in which the gauze was retained in the oral region was of 8 years (7), whereas in current case, this time was of 23 years before its removal. The main symptoms were reported in nearly every case [except one], in which the body’s reaction was to encapsulate the foreign body. In five cases, as in the current case report, symptoms of pain, swelling, and drainage of pus for long periods of time were reported.

Panoramic radiography, CT, magnetic resonance, and/or ultrasound contribute significantly to the diagnosis of gossypiboma. The radiographic features of gossypiboma are not well-defined. It could be observed that gossypiboma depends on the local reaction to the foreign body, the composition of the gauze, the containing or not of a radiopaque marker, and the retention time. These factors can also lead to misdiagnoses (2). In the cases of gossypiboma of the oral region and maxillary sinus, no images could be observed featuring radiopaque foreign bodies in the panoramic radiograph, but these did appear as well-demarcated radiolucencies (5). In the cases evaluated by CT, the image was described as an opacity in the maxillary sinus (4), determined by blind cavity fistulograma (7) or even a low-intensity, ill-defined mass with a spongiform aspect and irregular borders, containing air bubbles (6). The current case report demonstrated a radiopaque image, with irregular borders and some small radiolucent areas inside, due to the long period [23 years], which made the formation of dystrophic calcification possible.

In the oral region and maxillary sinus, there are several pathological processes with images and clinical symptoms that are similar to those caused by gossypiboma. According to the region and radiographic features found in cases in the literature, the differential diagnoses of the gossypiboma included: the recurrence of cysts in the maxillary region salivary fistula [submandibular], residual cysts or cystic ameloblastoma [retromolar region], and surgical wound infections [mandibular contour].

Histological examination is important in determining the diagnosis of gossypiboma. Sigron and Locher [2011] (5) reported a histopathology similar to that described in the current case report, that is, an aseptic chronic inflammatory infiltration with birefringent gauze fragments and dystrophic calcifications.

Treatment of the gossypiboma in the oral region consists of eliminating the inflammatory process by surgically removing the gauze, especially in symptomatic cases. Gossypiboma detected in other regions of the body sometimes it is left untreated, due to the absence of clinical symptoms (2). This conduct was not observed in the oral region. The treatment prognosis is generally quite good, as can be observed in all cases: when the causative factor was removed, the patient was cured.

Few case reports on gossypiboma after dental surgery can be found in English-language literature. Dental professionals need to pay closer attention when performing intraoperative procedures so as to minimize the chances of gauze retention in the oral region. Moreover, professionals should properly diagnose this disease, given that it commonly presents clinical signs and radiographic results that can easily be confused with other pathologies in the oral region.
